# 14,000-year-old seeds indicate the Levantine origin of the lost progenitor of faba bean

**DOI:** 10.1038/srep37399

**Published:** 2016-11-23

**Authors:** Valentina Caracuta, Mina Weinstein-Evron, Daniel Kaufman, Reuven Yeshurun, Jeremie Silvent, Elisabetta Boaretto

**Affiliations:** 1 Max Planck-Weizmann Center for Integrative Archaeology and Anthropology, 76100 Rehovot, Israel; 2 D-REAMS Radiocarbon Laboratory, 76100 Rehovot, Israel; 3 Zinman Institute of Archaeology, University of Haifa, Haifa 3498838, Israel; 4 Department of Structural Biology, Weizmann Institute of Science, 76100 Rehovot, Israel

## Abstract

The understanding of crop domestication is dependent on tracking the original geographical distribution of wild relatives. The faba bean (*Vicia faba* L.) is economically important in many countries around the world; nevertheless, its origin has been debated because its ancestor could not be securely identified. Recent investigations in the site of el-Wad (Mount Carmel, Israel), provide the first and, so far, only remains of the lost ancestor of faba bean. X-ray CT scan analysis of the faba beans provides the first set of measurements of the biometry of this species before its domestication. The presence of wild specimens in Mount Carmel, 14,000 years ago, supports that the wild variety grew nearby in the Lower Galilee where the first domestication was documented for Neolithic farmers 10,200 years ago.

Crop progenitor | Extinction| *Vicia faba* L.| Natufian| Radiocarbon Dating| X-ray CT scan.

The faba bean (*Vicia faba* L.) is one of the most cultivated legume crop that enhances soil productivity and offers a valid source of protein for humans and animals^1^. The faba bean’s ability to fix atmospheric nitrogen makes this legume useful in crop rotation and an important tool in reducing the concentration of the N_2_-greenhouse gas in the atmosphere. Despite its importance, little is known about the origin of the faba bean. All the living varieties are fully domesticated and neither wild representatives of this species nor any closely related species have been found^2^,^3^,^4^. Furthermore, the germplasm available within the domesticated forms of *Vicia faba* L. is inadequate to solve major problems such as susceptibility to various pests and diseases and tolerance to environmental stress^5^. In order to effectively solve these problems, new types of germplasm must be found^6^.

An essential factor in understanding the process of evolution of modern crops is the identification of the pre-domestication distribution of the wild progenitors. When humans started to select among wild plants those that had the most desirable features, they caused permanent alterations which, subsequently, made the plants unsuitable for survival in the wild and, in some cases, led to the extinction of the wild traits^7^. This process started around the11^th^ millennium BP in southwestern Asia, and accounts for the development of ‘domestication-traits’ such as the loss of dispersal mechanisms and reduced seed dormancy in edible plants^8^. Previous studies of archaeological faba bean, collected in the PPNB sites of Ahihud, Nahal Zippori and Yiftahel, provided insights about the timing of domestication of this crop and the geographic area where the first domestication occurred, but have not solved the enigma regarding the original area of distribution of its wild progenitor^9^,^10^,^11^. Prior to domestication, hunter-gatherers had acquired an intimate knowledge of plants and, at least by the end of the Pleistocene, they had diversified their diet by intensively gathering a wide range of plants, some of which would eventually become domesticated^12^. Thus, the study of the spectrum of plant species used by the last hunter-gatherers in southwestern Asia may offer new insights into the early history of edible plants, as well as provide invaluable information on extinct wild progenitors of modern crops.

Here we report the discovery of the wild type progenitor of faba beans in the prehistoric (Natufian) site of el-Wad Terrace (EWT), Mount Carmel, Israel. The site was inhabited by sedentary hunter-gathers who exploited a wide range of wild animals and plants^13^,^14^,^15^ several millennia before domestication.

Mount Carmel is a unique and highly diversified area, which encompasses the mountainous area of the Carmel, the coastal plain and the seashore (Fig. 1). Since 1996, it is a UNESCO biosphere, which hosts 976 of all the 1500 plant species of Israel^16^. The vegetation of the reserve includes over a hundred varieties of wild legumes and 10% of the varieties of wild *Vicia* ssp. documented in the world^17^. As such, Mount Carmel is one of the largest wild legume gene pools in western Asia. Mount Carmel is also known for providing prolific evidence regarding the Late Epi-paleolithic Natufian Culture (15,000–11,700 cal BP). The Natufian, renowned for the regular appearance of durable architecture, hewn bedrock features, cemeteries and art in the Levantine record, marks the pre-agricultural transition from nomadic to sedentary life-ways^18^. El-Wad (western Mount Carmel) displays a long Natufian cultural sequence upon which the definition of this culture was largely based^19^.

## Results

**Figure 2:**
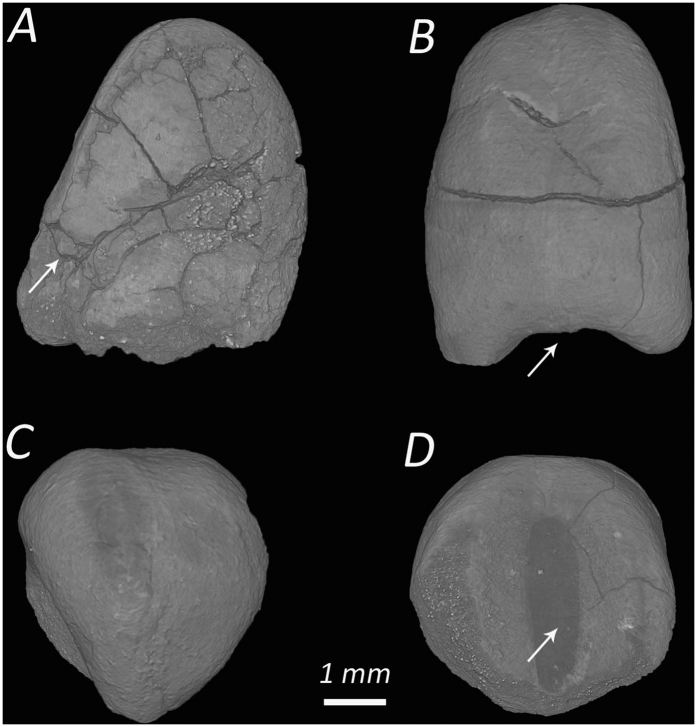
Micro-CT views of *Vicia faba* L. from EWT. (**A**) Lateral view of specimens O9d_B47 (Movie S1). The arrow points to the radicle which is only partially preserved; (**B**) dorsal view of specimen P7d_B123a (Movie S2). The arrow points to the bottom of the legume where the trace of the hilum was preserved; (**C**) top view of specimen P7d_B123b (Movie S3); (**D**) bottom view of P7d_B123a, the arrow points to the gray area in the middle where a trace of the hilum is preserved.

Recent excavation at EWT recovered plant remains from the earliest phase of occupation, namely the Early Natufian (~15,000–13,500 cal BP). Plant remains are rarely found in coeval sites in the southern Levant, so the study of the el-Wad material provides rare insights into the gathering and consuming of vegetal foods of the earliest occupants and their choices in selecting specific plants from the local wild stands. The plant material recovered at the site includes mostly hawthorn, wild barley, lentils and vetches (Supplementary Table S1). In addition, six medium-size legumes (~5 mm long) were found. Each of these is heart-shaped and presents two cotyledons and an elongated radicle that reaches the bottom of the seed. In one of the legumes, the negative traces of an oblong hilum are found near the bottom at the junction between the two cotyledons. While the heart-like shape of the legumes suggests that they likely belong to the Vicieae tribe, the elongated radicle and the oblong hilum at the bottom, prove that the legumes were, indeed, specimens of *Vicia faba* L. (Fig. 2). Comparison with modern specimens of domesticated varieties of faba beans, such and *Vicia faba* var. *minor* and *V. faba* var. *paucijuga*, confirms the identification. The position of the radicle and the hilum are unique to faba beans and are not found in other close wild relatives such as *Vicia narbonensis, V. johannis, V. palaestina* and *V. serratifolia*^20^,^21^,^22^.

### Radiocarbon dating

**Figure 3:**
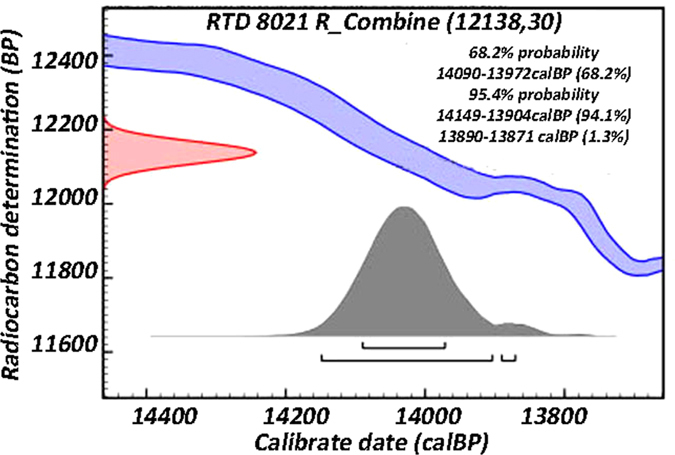
Calibrated date of faba bean from el-Wad Terrace. Probability distribution of the calibrated radiocarbon range of the faba sample RTD 8021.

The faba beans were found in an archaeological layer dated, on the basis of abundant cultural (lithic) material and radiocarbon measurements on charcoal and bones, to the Early Natufian (ca. 15,000–13,500 BP)^23^,^24^. One of the *Vicia faba* L. specimens (P7d_B123c) was radiocarbon dated twice (RTD-8021:12,145 ± 42 and 12131 ± 41 ^14^C years BP), providing an average date of 12,138 ± 30 ^14^C year BP (14,150-13,900 cal BP) (Fig. 3). This date is in agreement with the Early Natufian dates of the site. The dating of the EWT faba beans shows that this species was present in the local wild vegetation three millennia before the faba bean was domesticated^9^.

### Xray-CT scan and morphometric analysis

**Figure 4:**
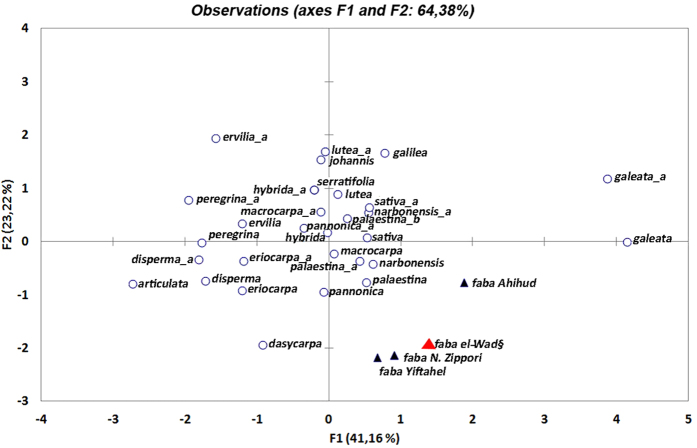
Plot of Principal Component Analysis (PCA) of the major Vicieae specimens. The plot shows analogies and differences between the faba bean from el-Wad (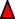), those from Ahihud, Nahal Zippori and Yiftahel (▲) and modern wild species native to Western Asia (○). The letters_a and_b are used to distinguish different seed and hilum outlines, within the same species. PCA axes 1 and 2 account for 65% of the cumulative variance. PCA axes represent respectively, the seed outline and the hilum relative length. For details, see Supplementary Table S3. Data for the modern species are from Zohary^17^, Gunn^20^ and Perrino *et al*.^21^. The plot was obtained using XLSTAT.

Biometric information, obtained by micro X-ray CT of three of the six specimens from EWT, provides the first set of data on the wild progenitor of faba bean (Supplementary Table S2). Using a Principal Component Analysis (PCA), the biometric data of EWT *Vicia faba* were compared with those available for wild species of *Vicia* ssp. natives of western Asia (Fig. 4). PCA enables direct quantitative comparison of the wild faba bean to other types of *Vicia*, highlighting similarities and differences between the wild progenitor and the known species of vetches. The variables considered are those usually employed in these kinds of studies such as seed length, seed circumference, seed outline, absolute hilum length, relative hilum length (as percentage of the seed circumference) and hilum outline. If we omit variables such as hilum and the radicle position that are in a unique location in *V. faba*, the wild progenitor of faba bean is morphometrically close to *V. narbonensis* and *V. palaestina.*

## Discussion

Rich archaeo-biological evidence is available from the Natufian sequence of el-Wad, pertaining to the diet and environments of these complex hunter-gatherers and contextualizing the faba finds. Studies of charred wood remains, pollen and phytoliths indicate that the Early Natufian hamlet flourished under a humid climate, in the context of oak woodlands. Evergreen oak and other typical eastern Mediterranean woody species were utilized, along with grasses^14^,^24^,^25^. Animal exploitation centered on hunting and probably trapping of a wide variety of mostly small-bodied game^13^,^26^. Together, the botanical and faunal data indicate a mosaic of open and wooded Mediterranean environments during the Natufian. Importantly, no evidence for plant or animal domestication is apparent at el-Wad (and other Natufian sites) with the notable exception of the dog^27^.

In light of the growing concern over the predicted devastating impact of climate change on global biodiversity and food security, identifying and preserving the wild progenitor has become a high priority. The wild progenitor of the crop could provide additional sources of genetic variability to improve the resilience of faba bean to biotic and abiotic stresses and to ensure more stable yields.

## Conclusion

Our discovery of faba beans in a sedentary hunter-gatherers campsite located only a few of kilometers away from the Lower Galilee, the area where the first domesticated faba bean were discovered^9^, date the earliest evidence of this legume to the 14^th^ millennium cal BP, almost three thousand years before its domestication. The discovery points to one of the possible areas where the wild progenitor of faba bean grew and offers new insights into the ecological requirements of the wild relative. Conceivably, a wild type faba bean would grow in a habitat which, today, resembles that of Mount Carmel 14,000 years ago. Given that each plant species represents a genetic group that developed adapting itself to a definite environment, identifying the original geographical distribution of the wild faba is fundamental to understanding the process of evolution of the species and the ecological requirements of its wild ancestor.

## Methods

### Archaeobotanical analysis

The plant remains presented in this paper come from eight 1 m squares from elevations ranging between 330 and 450 cm below datum. All the sediments collected were wet sieved using meshes of 5 mm and 1mm. Once the samples were dried, charred seeds were sorted using a binocular microscope (Olympus SZ51). A reference collection of modern wild seeds, provided by the Israel Gene Bank for Agricultural Crops of the Volcani Center, Bet-Dagan, was used to identify the archaeological seeds.

### Micro X-ray Computer Tomography (X-ray CT)

Image analysis of three of the six archaeological faba beans found in EWT was performed using a micro X-ray Computed Tomography. X-ray CT allowed a non-destructive volume visualization of the three faba beans, respectively labeled as O9a-Basket 47, P7d-Basket 123a and b. The seeds were placed in a parafilm-sealed 1 ml pipette tip, which was installed in the standard sample holder. The seeds were analyzed using a Xradia Micro-CT-400 (Zeiss X-Ray Microscopy, Pleasanton, CA, USA), with a X-ray source of 30 kV, current of 150 μA and 0.47617X magnification, and then processed using the software Avizo 7 (FEI Visualization Sciences Group).

Measurements were taken with a pixel size resolution of 5.7975 μm and 1,000 projection images for specimen O9d-Basket 47, and pixel size of 6.6234 μm with 1,000 and 1,200 projections images, respectively, for P7d-Basket 123a and b. The images were recorded with exposure times of five seconds while the seeds rotated over 180 degrees. Computer-processed combinations of many images were taken from different angles to produce cross-sectional images (virtual ‘slices’) of the scanned seeds. The ‘virtual slices’ were processed using mathematical algorithms to infer quantitative information on the shape and morphometry of each seed using Avizo 7 (FEI Vizualization Sciences Group) software. All the acquisitions were done in the Department of Chemical Research Support, Weizmann Institute of Science, Rehovot, Israel.

The 3D models of the three seeds are included as videos in the supplementary material, and they are used for disseminative purposes on the publication associated to the project.

### Biometric Analysis

The biometric analysis of the EWT legumes was performed using X-ray Computer Tomography in order to get the most accurate set of measurements. The parameters selected for measuring are those commonly used to discriminate among different species and subspecies of Vicieae such as seed length, seed circumference and seed outline. In addition, absolute hilum length, relative hilum length (as % of the seed circumference) and hilum outline were inferred from the hilum fingerprint on the cotyledons. In order to provide the most complete set of biometric data on the seeds, measurements of seed volume, width and position of the radicle were also taken. Based on a previous study that analyzed the morphological changes of *V. faba* combusted at different temperatures over different time periods^9^, we were able to estimate the original size of the archaeological faba bean. The estimated circumference is reported next to the value of real circumference in Supplementary Table S2.

### Principal Component Analysis (PCA)

The parameters used in this work were already described in previous studies. All the measures presented in Supplementary Table S3 were obtained by Gunn^20^, Perrino *et al*.^21^, Chernoff *et al*.^22^. The parameters considered are those commonly adopted for the classification of the seeds, such as seed maximum circumference and seed outline. Other parameters are those used to describe the hilum: hilum absolute length, hilum relative length and hilum outline. While some of these parameters, such as seed circumference and hilum length are quantitative measures of anatomical features, others, such as seed outline, relative hilum length and hilum outline are qualitative parameters.

The classification of the seed outline used in this study is based on six types: 1 = heart-shaped (pyriform); 2 = sub-triangular; 3 = ovate; 4 = sub-lenticular; 5 = sub-spherical; 6 = sub-rectangular. Relative hilum length is the percentage of the seed circumference occupied by the hilum. According to Gunn^20^, there are six categories of relative hilum length: very long (80–70%), long (70–60%), medium long (60–50%), medium short (50–30%), short (20–10%), very short (below 10%). With the exception of *V. galeata*, the majority of the Vicieae analyzed in this study belong to the short and very short categories. The hilum outline can vary in shape and portion of the seed circumference occupied. Scholars identified five categories of hilum outline: 1 = circumlinear, 2 = linear, 3 = oblong, 4 = wedge, 5 = oval^20^,^21^. For the subspecies of Vicieae considered in this study, only four categories of hilum outline are represented: 2 = linear, 3 = oblong, 4 = wedge, 5 = oval.

### Radiocarbon dating of archaeological material

The sample was pre-treated, graphitized and measured by Accelerator Mass Spectrometry at D-REAMS Radiocarbon Laboratory of the Weizmann Institute of Science, Israel. The legume (~30 mg of material) was cleaned using Acid-Base-Acid treatment as in Yitzhaq *et al*.^28^. The sample prepared for dating was combusted to CO_2_ in quartz tubes containing about 200 mg of copper oxide (Merck) and heated to 900 °C for 200 min. The CO_2_ was divided into three aliquots and each was reduced to graphite using cobalt (Fluka) (about 1 mg) as a catalyst and hydrogen at 700 °C for 20 hr. The ^14^C ages were calibrated to calendar years BP and BC using the IntCal13 atmospheric curve^29^ using the software OxCal v 4.2.3^30^.

## Additional Information

**How to cite this article**: Caracuta, V. *et al*. 14,000-year-old seeds indicate the Levantine origin of the lost progenitor of faba bean. *Sci. Rep.* **6**, 37399; doi: 10.1038/srep37399 (2016).

**Publisher's note:** Springer Nature remains neutral with regard to jurisdictional claims in published maps and institutional affiliations.

## Supplementary Material

Supplementary Material

Supplementary Video 1

Supplementary Video 2

Supplementary Video 3

## Figures and Tables

**Figure 1:**
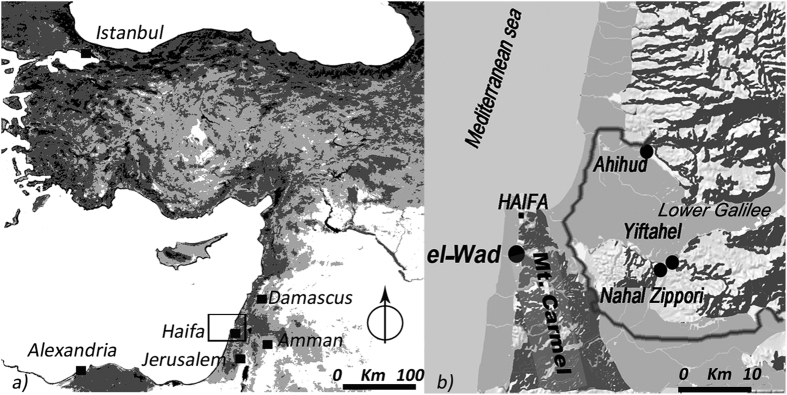
The context of study. (**a**) Map of the Near East; (**b**) Location of the Natufian site of el-Wad and the Pre-Pottery Neolithic B sites of Ahihud, Yiftahel, and Nahal Zippori where the earliest domesticated faba beans were found^9^,^10^. In grey the limit of the Lower Galilee. The images were created with QGIS Development Team, <2015>. QGIS Geographic Information System. Open Source Geospatial Foundation Project. http://www.qgis.org/.
