# Supplementary material for: 14,000-year-old seeds indicate the Levantine origin of the lost progenitor of faba bean
Source: Sci Rep. 2016 Nov 23;6:37399. doi: 10.1038/srep37399 (PMC5120295; doi:10.1038/srep37399)
Supplement: Supplementary Material [file srep37399-s1.doc]

**14,000-years-old seeds indicate the Levantine origin of the lost progenitor of faba bean.**

Valentina Caracuta, Mina Weinstein-Evron, Daniel Kaufman, Reuven Yeshurun, Jeremie Silvent and Elisabetta Boaretto.

**Supplementary Information**

**Archaeology**. The Natufian Culture, culminating the Epipaleolithic cultural sequence of the terminal Pleistocene southern Levant (ca. 15,000–11,700 cal. BP) is widely thought to reflect a relatively complex and sedentary hunter-gatherer adaptation, just preceding the transition to agriculture in southwest Asia. This view is based on the appearance during this period of large sites with durable architecture and hewnbedrock features, a rich groundstone industry and artistic repertoire, evidence for economic intensification, the presence of commensal animals, and cemeteries exhibiting elaborate burial customs (18). Following the Natufian, plant and animal domestication coupled with the rise of villages and unprecedented social complexity are evidenced in the same region during the Pre-Pottery Neolithic period.

One of the major Natufian base-camps exhibiting all of its characteristic features, the el-Wad Cave, is part of the UNESCO World Heritage Site complex of Nahal Me‛arot/Wadi el-Mughara that also includes the caves of Tabun, Jamal and Skhul (19).The site, a large cave with an adjacent terrace containing a long and rich Early, Late and Final Natufian sequence, is situated on the western face of Mount Carmel, Israel, where the mountain cliff meets the open expanses of the Mediterranean coastal plain, ca. 45 m above modern sea level, became well-known as a result of Garrod’s 1929–1933 excavation campaign (19). Garrod’s finds from el-Wad were the foundation for her subsequent definition and interpretation of the Natufian culture as a transitional phase between foraging and fully agricultural life-styles. The renewed and on-going excavation was initiated in 1994 and has focused on the north-eastern part of the terrace. An area of ca. 70 m^2^ has been exposed and the attained thickness of Natufian sediments ranges between ca. 0.5 m to 1.5 m. Structures, burials and a high density of finds were retrieved during the renewed excavation (13,31).

A composite stratigraphy of the site, based on a compilation of data from all excavations, suggests an ephemeral occupation at the base of the Early Natufian (designated Early EarlyNatufian or EEN), followed by a prolific burial phase comprising almost 100 individuals (Middle Early Natufian or MEN) and culminating with the Late Early Natufian (LEN). This phase appears as a massive, >0.5 m thick accumulation of repeated occupations. The renewed excavation has exposed an architectural complex in the LEN phase (**Fig. S1**) composed of a nine meter long curvilinear wall (Wall I) encompassing a sequence of at least eleven architectural levels, each defined by a thin stony floor. In the area enclosed by Wall I, several partially preserved stone structures and stone-rich ‘living floors’ have been excavated. Overlying this architectural phase are thick Early Natufian living levels with a few stone features but normally lacking structures. The Natufian sequence ends with a thinner Late Natufian layer devoid of architecture, but displaying several installations and concentrations of graves (4). The Early Natufian occupations (Garrod’s layer B2, our Unit 2, Phases W-7 to W-3/2) are much thicker and richer than the Late and Final Natufian layers throughout the site (Garrod’s layer B1; here, the upper part of Unit II [Phases W-2/1 to W-0] and base of Unit I).

The Natufian occupations at el-Wad have been dated by several radiocarbon series. The recent el-Wad Terrace (EWT) excavation (where the material in the present study originates) provided thirteen radiocarbon measurements on charcoals and ungulate bones to yield a calibrated age range of 14,950–13,950 years BP (±2σ) for the architecture-bearing phases and a range of ca. 15,000–13,000 years BP (±2σ) for the entire Unit II accumulation of the terrace (31). Additionally, a section in square O5 **(Fig.S1)** that was excavated in 2012 gave a series of dates covering the Early Natufian to modern occupations of the terrace. The Early Natufian layer was dated to 14,705–13,195 years BP (±2σ) and, more specifically, the architecture-bearing phase within this layer was dated to 14,705–13,820 years BP (±2σ). The Late Natufian layer provided dates of 13,765–11,835 years BP (±2σ) and later (ephemeral occupations of the terrace produced Holocene dates of 9,285–1,950 years BP (±2σ) (24). Thus, the direct dates obtained from the faba seeds place them securely in the Early Natufian layer of the site.

The faba seeds discussed here come from the earliest attained phase in the NE Terrace excavation, Phase W-7, which is a thick Early Natufian phase displaying repeated building of walls and floors, within a large architectural compound demarcated by a curvilinear terrace wall (Wall I; **see plan in Fig. S1**). The seeds were associated with numerous remains which were deposited inside the Wall I complex, including lithics, faunal remains, minerals and shells. Importantly, numerous fragments of pestles and some other grinding gear, made of basalt and limestone, as well as sickle blades made of flint were found in this phase, and others, attesting to intensive plant collection and processing. So far, six whole faba beans and one-half bean were collected from the EN layers excavated in EWT. These were retrieved from the grid squares O7, O9 and P7 (**Fig. S1;**for provenance details see **Table S1).**

**Archaeobotanical analysis**

The archaeobotanical analysis allowed for the identification of more than fifty seeds. The Minimum Number of Individuals (MNI) was estimated by adding *n* half/2 +*n* fragments/4. For example, if 1 *Hordeum* sp. grain was found together with 4 halves and 19 fragments, the MNI estimated was 1+4/2+19/4=8.

As a result, we can assume that at least 52 seeds can be ascribed to the EN phase. The majority are wild legumes, followed by wild cereals, fruit-tree stones and shells and weeds (**Table S1**). Most of the legumes are *Lens* sp. (lentil) and *Vicia faba* (faba bean), while only a few are *Vicia palaestina* and *Vicia peregrina*. The cereals are exclusively grains of *Hordeum spontaneum* (wild barley) and *Hordeum* sp. Remains of wild almonds (*Amygdalus* sp. fruit-shells) and hawthorn fruits (*Crataegus* sp. stones) are also part of the archaeobotanical assemblage of EWT. The weeds identified are *Cirsium* sp., *Bellevalia* sp., *Brassica* sp. and *Ornithogalum* sp. While the first does not have any known use, *Bellevalia* and *Ornithogalum* species, both of the Asparagaceae family, haves succulent edible bulbs that are commonly consumed boiled or roasted. *Brassica* subspecies are renowned for their edible values; members of this species are broccoli, cabbage, cauliflower and mustard. The plant species identified suggest that a wide range of wild taxa were available to the Natufian inhabitants of EWT during the Early Natufian period. Of such species, at least four (i.e. lentil, faba beans, barley and almond) eventually became domesticated.

**Biometric Analysis.** Like seeds of most other legumes, seeds of Vicieae have an external coat which consists of a testa, hilum, lens and micropyle and an internal embrio composed of two cotyledons and an axis with a radicle. External and internal morphological characters of Vicieae have been used to identify species and distinguish among subspecies (20), but these studies were aimed at discriminating fresh modern seeds where both external and internal morphological characters are preserved. Archaeological seeds, in most cases, lack the external coat so the analysis of morphometric traits is limited to the cotyledons, the radicle and, wherever is preserved, to the fingerprint of the hilum on the cotyledons.

**Principal Component Analysis (PCA)** was adopted to emphasize variations and bring out strong patterns in the dataset of modern and ancient specimens of Vicieae. The ultimate goal was to highlight existing correlations between modern varieties of Vicieae and the archeological specimens of *V. faba*. Previous studies, conducted on the modern varieties of Vicieae (21,22), provide a database of biometrical parameters used as comparison for the study of *V. faba* found in EWT. Overall, thirty modern specimens, representing seventeen botanical varieties of wild Vicieae growing in the eastern Mediterranean, were chosen.


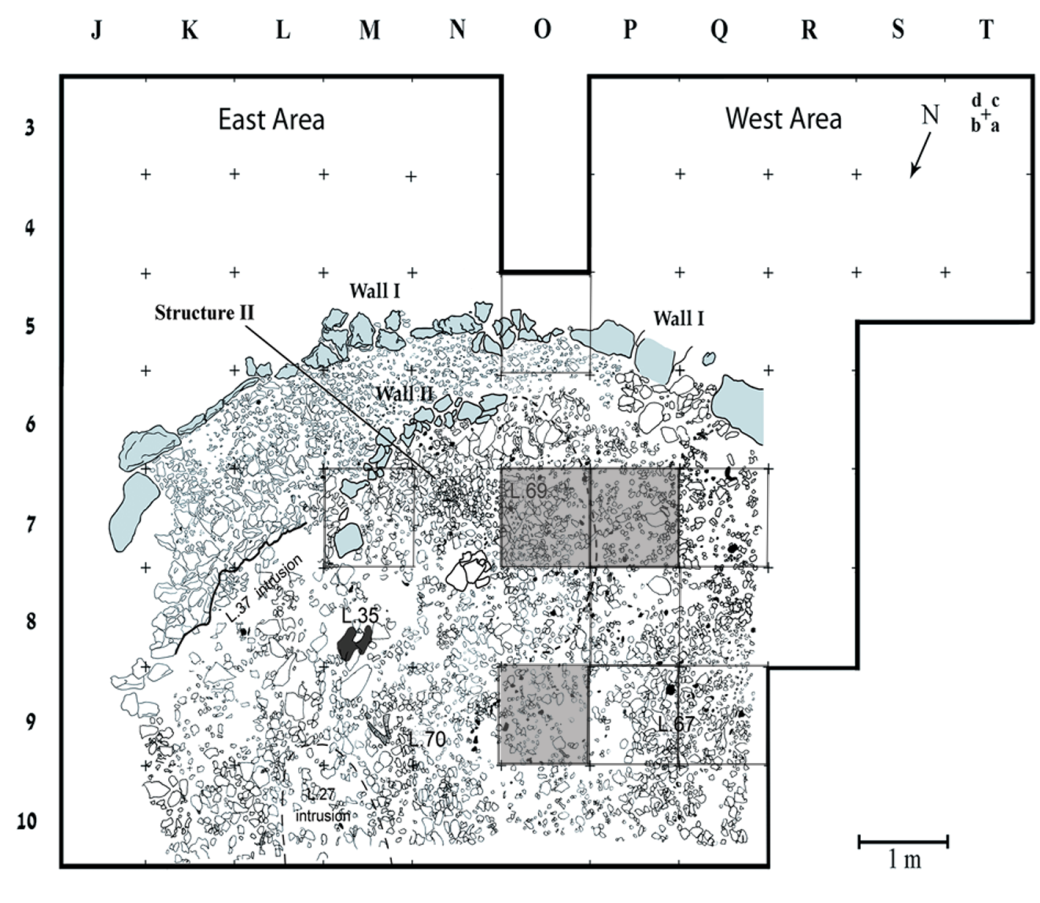


**Figure S1| Plan of the EWT excavation**. the Early Natufian architectural complex is shown. Unshaded squares indicate the squares from which the archaeobotanical material was collected. Shaded squares indicate where the *Vicia faba* was found. The original version of the map was hand-draw by Dr. Yesherun. The image was computerized using Adobe Photoshop CS5 <http://www.adobe.com/products/photoshop.html> (Copyright holder Reuven Yeshurun).

**Table S1| Plant remains from the Early Natufian Layers in EWT. Information on the provenience of the sample: square, sub-square, locus, basket and elevation. The volume of sediment is expressed in Liters (Lt) unless it has not been recorded (NR). Species of plant are grouped in categories: fruit-trees, legumes, cereals, weeds. The number of plant remains is given for each species and each sample. Fragments are reported as (fr) and half as (h).**

|  | | | | | | Fruit Trees | | | Legumes | | | | | | | | | Cereals | | | | Weeds | | | | |
| --- | --- | --- | --- | --- | --- | --- | --- | --- | --- | --- | --- | --- | --- | --- | --- | --- | --- | --- | --- | --- | --- | --- | --- | --- | --- | --- |
| Square | Sub.Square | Locus | Basket | Elevation (cm) | Volume (Lt) | *Amygdalus* sp. (shell) | *Crataegus* sp. | Unidentified fruit-shell | Leguminosae small (>2mm) | Leguminosae medium (>5mm) | *Lens* sp. | *Lathyrus apacha* | *Vicia* sp. | *Vicia faba* | *Vicia palaestina* | *Vicia peregrina* | *Vicia sativa/Pisum* sp. | *Hordeum spontaneum* | *Hordeum sp.* | *Triticum dicoccoides* | *Poacea* | *Cirsium sp.* | *Bellevalia* sp. | *Brassica sp.* | *Onithogalum sp.* | Ind. Small seed(>2mm) |
| M9 | a | 79 | 79 | 465-466 | NR |  |  | 1fr |  |  |  |  |  |  |  |  |  |  |  |  |  |  |  |  |  |  |
| O5 | a |  | 64 | 312-317 | 2,00 |  | 4fr |  |  |  |  |  |  |  |  |  |  |  |  | 1 |  |  |  |  |  |  |
| O5 | a |  | 69 | 317-322 | 1,67 |  |  |  |  |  |  |  |  |  |  |  |  |  | 1fr |  |  |  |  |  |  |  |
| O5 | d |  | 70 | 318-323 | 2,50 |  |  |  |  | 1fr |  |  |  |  |  |  |  |  |  |  |  |  |  |  |  |  |
| O5 | d |  | 73 | 323-328 | 2,33 |  | 1fr |  |  |  |  |  |  |  |  |  |  | 1 |  |  |  |  |  |  |  |  |
| O5 | a |  | 76 | 326-332 | 2,33 |  |  |  |  |  |  |  |  |  |  |  |  |  | 2fr |  |  |  |  |  |  |  |
| O5 | d |  | 77 | 328-333 | 2,50 |  |  |  |  |  |  |  |  |  |  |  | 1 | 1h |  |  |  |  |  |  |  |  |
| O5 | c |  | 78 | 323-346 | 2,33 |  |  |  |  |  |  |  |  |  |  |  |  | 1 |  |  |  |  |  |  |  |  |
| O5 | b |  | 79 | 331-336 | 2,50 |  |  |  |  |  |  |  |  |  |  |  |  | 1 |  |  |  |  |  |  |  |  |
| O5 | b |  | 81 | 336-341 | 2,00 |  |  |  |  |  |  |  | 1h |  |  |  |  | 1 |  |  |  |  |  |  |  |  |
| O5 | a |  | 82 | 332-337 | 2,00 |  |  |  |  |  |  |  |  |  |  |  |  |  | 1h |  |  |  |  |  |  |  |
| O5 | d |  | 83 | 333-339 | 2,25 |  |  |  | 1h |  |  |  |  |  |  |  |  |  |  |  |  |  |  |  |  |  |
| O5 | a |  | 85 | 337-340 | 1,50 |  |  |  |  | 1h |  |  |  |  |  |  |  |  | 1+1h |  |  |  |  |  |  |  |
| O5 | b |  | 88 | 341-345 | 1,50 |  |  |  | 1fr |  |  |  |  |  |  |  |  |  |  |  |  |  |  |  |  |  |
| O5 | a |  | 89 | 340-345 | 1,50 |  |  |  |  |  |  |  |  |  |  |  |  |  | 2fr |  |  |  |  |  |  |  |
| O5 | b |  | 90 | 345-350 | 2,50 |  | 3fr |  |  |  |  |  |  |  |  |  |  |  | 1h+2fr |  |  |  |  |  |  |  |
| O5 | a |  | 95 | 340-350 | 1,50 |  |  |  | 3fr |  |  |  |  |  |  |  |  |  |  |  |  |  |  |  |  |  |
| O5 | a |  | 96 | 350-355 | 1,33 |  |  |  | 2fr |  | 1 |  |  |  |  |  |  |  |  |  |  |  |  |  |  |  |
| O5 | b |  | 97 | 335-360 | 1,13 |  |  |  |  |  | 1 |  |  |  |  |  |  | 1h |  |  |  |  |  |  |  |  |
| O5 | a |  | 99 | 355-361 | 1,00 |  | 4fr |  |  |  |  |  |  |  |  |  |  |  |  |  |  |  |  |  |  |  |
| O5 | a | 72 | 102 | 352-359 | 0,50 |  |  |  | 1 |  |  |  |  |  |  |  |  |  |  |  |  |  |  |  | 6 |  |
| O5 | a |  | 109 | 369-373 | 1,00 |  |  |  |  |  |  |  | 1h |  |  |  |  |  |  |  |  |  |  |  |  |  |
| O5 | a |  | 113 | 373-377 | 1,33 |  |  |  | 1h |  |  |  |  |  |  |  |  |  |  |  |  |  |  |  |  |  |
| O5 | b |  | 114 | 374-379 | 1,00 |  | 1 |  |  |  | 1h |  |  |  |  |  |  |  |  |  |  |  |  |  |  |  |
| O5 | b |  | 116 | 379-384 | 1,00 |  | 3fr |  |  | 1 |  |  |  |  |  |  |  |  |  |  |  |  |  |  |  |  |
| O5 | b |  | 120 | 385-389 | 1,50 |  |  |  |  | 1fr |  |  |  |  |  |  |  | 1 |  |  |  |  |  |  |  | 1 |
| O5 | a |  | 123 | 388-405 | 0,20 |  |  |  |  |  |  |  |  |  |  |  |  |  | 1h |  |  |  |  |  |  |  |
|  |  |  |  |  |  |  |  |  |  |  |  |  |  |  |  |  |  |  |  |  |  |  |  |  |  |  |

**Continues from previous page. Table S1**

|  | | | | | | Fruit Trees | | | Legumes | | | | | | | | | Cereals | | | | Weeds | | | | |
| --- | --- | --- | --- | --- | --- | --- | --- | --- | --- | --- | --- | --- | --- | --- | --- | --- | --- | --- | --- | --- | --- | --- | --- | --- | --- | --- |
| Square | Sub.Square | Locus | Basket | Elevation (cm) | Volume (Lt) | *Amygdalus* sp. (shell) | *Crataegus* sp. | Unidentified fruit-shell | Leguminosae small (>2mm) | Leguminosae medium (>5mm) | *Lens* sp. | *Lathyrus apacha* | *Vicia* sp. | *Vicia faba* | *Vicia palaestina* | *Vicia peregrina* | *Vicia sativa/Pisum* sp. | *Hordeum spontaneum* | *Hordeum sp.* | *Triticum dicoccoides* | *Poacea* | *Cirsium sp.* | *Bellevalia* sp. | *Brassica sp.* | *Onithogalum sp.* | Ind. Small seed(>2mm) |
| O7 | a | 62 | 91 | 401-405 | 1,00 |  |  |  | 3fr |  |  |  |  |  |  |  |  |  |  |  |  |  |  |  |  |  |
| O7 | c |  | 94 | 399-403 | 0,75 |  |  |  |  | 1h |  |  |  |  |  |  |  |  |  |  |  |  |  |  |  |  |
| O7 | a |  | 95 | 405-405 | 0,67 |  |  |  |  | 1h |  |  |  |  |  |  |  |  |  |  |  |  |  |  |  |  |
| O7 | a |  | 102 | 407-410 | 0,50 |  |  |  |  | 1fr |  |  |  |  |  |  |  |  |  |  |  |  |  |  |  |  |
| O7 | a |  | 113 | 413-415 | 0,50 |  |  |  | 1fr |  |  |  |  |  |  |  |  | 1 |  |  |  |  |  |  |  |  |
| O7 | b |  | 115 | 419-420 | 0,33 |  |  |  |  | 1h |  |  |  |  |  |  |  |  |  |  |  |  |  |  |  |  |
| O7 | c |  | 116 | 407-408 | 0,20 |  |  |  |  | 1h |  |  |  |  |  |  |  |  |  |  |  |  |  |  |  |  |
| O7 | b |  | 119 | 420-422 | 0,50 |  |  |  | 2fr |  |  |  |  |  |  |  |  |  |  |  |  |  |  |  |  |  |
| O7 | c |  | 120 | 408-411 | 0,75 |  |  |  | 2fr | 2fr |  |  |  |  |  |  |  |  |  |  |  |  |  |  |  |  |
| O7 | a |  | 121 | 415-416 | 0,33 |  | 1fr |  |  |  |  |  |  |  |  | 1h |  |  |  |  |  | 1 |  |  |  |  |
| O7 | c | 69 | 122 | 411-411 | 0,25 |  |  |  |  |  | 1 |  |  |  |  |  |  |  |  |  |  |  |  |  |  |  |
| O7 | b | 78 | 128 | 423-426 | 0,50 |  |  |  | 1fr |  |  |  | 1 |  |  |  |  |  |  |  |  |  |  |  |  |  |
| O7 | a | 78 | 129 | 417-418 | 0,25 |  |  |  |  |  |  |  |  |  |  |  |  | 1 |  |  |  |  |  |  |  |  |
| O7 | c | 78 | 130 | 414-416 | 0,50 |  |  |  |  |  |  |  |  | 1 | 1h |  |  |  |  |  |  |  |  |  |  |  |
| O7 | d | 78 | 131 | 418-423 | 0,50 |  |  |  |  |  | 1 |  |  |  |  |  |  | 1h |  |  |  |  |  |  |  |  |
| O7 | d |  | 132-1 | 423-424 | 0,25 |  |  |  |  | 1h |  |  |  |  |  |  |  |  |  |  |  |  |  |  |  |  |
| O7 | b |  | 133 | 426-433 | 1,00 |  |  |  |  | 1h |  |  |  |  |  |  |  |  |  |  |  |  |  |  |  |  |
| O9 | d |  | 42 | 420-426 | 2,75 |  | 6fr |  |  |  |  |  |  |  |  |  |  |  |  |  |  |  |  |  |  |  |
| O9 | a |  | 47 | 424-428 | 3,00 |  |  |  |  |  |  |  |  | 1 |  |  |  | 2 |  |  |  |  |  |  |  |  |
| O9 | c |  | 62 | 432-436 | 1,33 |  |  |  |  |  |  |  |  |  |  |  |  |  |  |  |  |  |  |  |  | 1 |
| O9 | b |  | 61 | 446-447 | 1,00 |  |  |  |  | 1h+8fr |  |  |  |  |  | 1 |  |  |  |  |  |  |  |  |  |  |
| O9 | d |  | 60 | 444-448 | 1,75 |  |  | 1fr |  | 1fr |  |  |  |  |  |  |  |  | 1fr |  |  |  |  |  |  |  |
| O9 | a |  | 64 | 443-445 | 0,25 |  |  |  |  | 1h+1fr |  |  |  |  |  |  |  |  |  |  |  |  |  |  |  |  |
| O9 | c |  | 45 | 419-424 | NR |  |  |  |  | 1fr |  |  |  |  |  |  |  | 1 |  |  |  |  |  |  |  |  |
| O9 | b |  | 46 | 431-434 | 2,00 |  |  |  | 1h |  |  |  |  |  |  |  |  |  |  |  |  |  |  |  |  |  |
| O9 | c |  | 50 | 424-427 | 1,00 |  |  |  | 2fr | 3fr |  |  |  |  |  |  |  |  |  |  |  |  |  |  |  |  |

**Continues from previous page. Table S1**

|  | | | | | | Fruit Trees | | | Legumes | | | | | | | | | Cereals | | | | Weeds | | | | |
| --- | --- | --- | --- | --- | --- | --- | --- | --- | --- | --- | --- | --- | --- | --- | --- | --- | --- | --- | --- | --- | --- | --- | --- | --- | --- | --- |
| Square | Sub.Square | Locus | Basket | Elevation (cm) | Volume (Lt) | *Amygdalus* sp. (shell) | *Crataegus* sp. | Unidentified fruit-shell | Leguminosae small (>2mm) | Leguminosae medium (>5mm) | *Lens* sp. | *Lathyrus apacha* | *Vicia* sp. | *Vicia faba* | *Vicia palaestina* | *Vicia peregrina* | *Vicia sativa/Pisum* sp. | *Hordeum spontaneum* | *Hordeum sp.* | *Triticum dicoccoides* | *Poacea* | *Cirsium sp.* | *Bellevalia* sp. | *Brassica sp.* | *Onithogalum sp.* | Ind. Small seed(>2mm) |
| P7 | c |  | 121 | 399-402 | 0,67 |  |  |  | 1fr |  |  |  |  |  |  |  |  |  | 1fr |  |  |  |  |  |  |  |
| P7 | c |  | 118 | 398-399 | 0,66 |  |  |  | 1fr |  |  |  |  |  |  |  |  |  |  |  |  |  |  |  |  |  |
| P7 | c |  | 123 | 402-407 | 2,00 |  |  |  |  | 1h+2fr | 1+1h |  |  | 3 |  |  |  |  |  |  |  |  |  |  |  |  |
| P7 | d |  | 122 | 404-408 | 1,30 |  |  |  |  | 1h+2fr |  |  |  |  |  |  |  | 1h |  |  |  |  |  |  |  |  |
| P7 | b |  | 119 | 405-407 | 1,00 |  | 3fr |  |  |  |  |  |  |  |  |  |  | 1h |  |  |  |  |  |  |  |  |
| P7 | a |  | 124 | 407-413 | 1,50 | 1fr | 1fr |  |  | 5fr |  |  |  | 1 |  |  |  | 1h |  |  |  |  |  |  |  |  |
| P7 | b |  | 120 | 404-412 | 1,10 |  |  |  | 1fr |  |  |  |  |  |  |  |  | 1h | 1fr |  |  |  |  |  |  |  |
| P7 | d |  | 124 | 408-411 | 2,00 |  | 1h+3fr |  |  | 16fr |  |  |  | 1h |  |  |  |  |  |  |  |  |  |  |  |  |
| P7 | d | 78 | 128 | 411-411 | 0,25 |  |  |  |  |  |  |  | 1 |  |  |  |  |  |  |  |  |  |  |  |  |  |
| P7 | d |  | 129 | 411-415 | 1,00 |  |  |  |  | 1h |  |  |  |  |  |  |  |  |  |  |  |  |  |  |  |  |
| P7 | b |  | 125 | 412-413 | 1,00 |  | 4fr |  |  |  | 2 |  |  |  |  |  |  |  |  |  |  |  |  |  |  |  |
| P7 | d |  | 131 | 419-423 | 0,50 |  | 3fr |  |  |  |  |  |  |  |  |  |  |  |  |  |  |  |  |  |  |  |
| P8 | c |  | 109 | 416-417 | 0,50 |  |  |  | 2fr |  |  |  |  |  |  |  |  |  |  |  |  |  |  |  |  |  |
| P8 | d |  | 110 | 412-417 | 1,50 |  |  |  |  | 2fr |  |  |  |  |  |  |  |  |  |  |  |  | 1 |  |  |  |
| P9 | a |  | 63 | 423-424 | 0,33 |  |  |  | 1fr |  |  |  |  |  |  |  |  |  |  |  |  |  |  |  |  |  |
| P9 | d |  | 71 | 437-438 | 0,50 |  |  |  |  |  | 1h |  |  |  |  |  |  |  |  |  |  |  |  |  |  |  |
| Q7 | c |  | 65 | 327-332 | NR |  |  |  |  | 1 | 1 | 1 |  |  |  |  |  | 16 |  |  |  |  |  |  |  |  |
| Q7 | d |  | 67 | 333-340 | NR |  |  | 10 fr |  |  |  |  |  |  |  |  |  |  | 4fr |  | 1 |  |  |  |  |  |
| Q7 | d |  | 76 | 344-349 | NR |  | 1 |  |  |  |  |  |  |  |  |  |  |  |  |  |  |  |  |  |  |  |
| Q7 | a |  | 78 | 353-353 | NR |  |  |  | 2+1h |  | 1 |  |  |  |  |  |  | 3 |  |  |  |  |  |  |  |  |
| Q7 | a |  | 78 | 353-353 | NR |  |  |  | 1fr |  |  |  |  |  |  |  |  |  |  |  |  |  |  | 1 |  |  |
| Q7 | a |  | 81 | 359-358 | NR |  | 1 |  |  |  |  |  |  |  |  |  |  |  |  |  |  |  |  |  |  |  |
| Q7 | b |  | 82 | 354-360 | NR |  |  |  |  | 1 |  |  |  |  |  |  |  | 1 | 5fr |  |  |  |  |  |  |  |
| Q7 | c |  | 140 | 405-406 | 1,33 |  | 1 |  |  |  |  |  |  |  |  |  |  |  |  |  |  |  |  |  |  |  |
| Q9 | d |  | 47 | 398-404 | 1,75 | 3fr |  |  | 3fr |  |  |  | 1 |  |  |  |  | 1 |  |  |  |  |  |  |  |  |
| Q9 | c |  | 63 | 402-408 | 0,75 |  | 2fr |  |  |  |  |  |  |  |  |  |  |  |  |  |  |  |  |  |  |  |

**Continues from previous page. Table S1**

|  | | | | | | Fruit Trees | | | Legumes | | | | | | | | | Cereals | | | | Weeds | | | | |
| --- | --- | --- | --- | --- | --- | --- | --- | --- | --- | --- | --- | --- | --- | --- | --- | --- | --- | --- | --- | --- | --- | --- | --- | --- | --- | --- |
| Square | Sub.Square | Locus | Basket | Elevation (cm) | Volume (Lt) | *Amygdalus* sp. (shell) | *Crataegus* sp. | Unidentified fruit-shell | Leguminosae small (>2mm) | Leguminosae medium (>5mm) | *Lens* sp. | *Lathyrus apacha* | *Vicia* sp. | *Vicia faba* | *Vicia palaestina* | *Vicia peregrina* | *Vicia sativa/Pisum* sp. | *Hordeum spontaneum* | *Hordeum sp.* | *Triticum dicoccoides* | *Poacea* | *Cirsium sp.* | *Bellevalia* sp. | *Brassica sp.* | *Onithogalum sp.* | Ind. Small seed(>2mm) |
| Q9/P9 | a |  | 60 | 416-421 | 1,50 |  | 1fr |  | 1 |  |  |  |  |  |  |  |  |  |  |  |  |  |  |  |  |  |
| Q9 | c |  | 64 | 408-408 | 0,20 |  | 1fr |  |  |  |  |  |  |  |  |  |  |  |  |  |  |  |  |  |  |  |
| Total | | | | |  |  | 5 |  | 4 | 3 | 9 | 1 | 3 | 6 |  | 1 | 1 | 31 | 1 | 1 | 1 | 1 | 1 | 1 | 6 | 2 |
|  |  |  |  |  |  |  | 1h |  | 4h | 12h | 3h |  | 2h | 1h | 1h | 1h |  | 7h | 4h |  |  |  |  |  |  |  |
|  |  |  |  |  |  | 4 fr | 40fr | 12fr | 26fr | 46fr |  |  |  |  |  |  |  |  | 19fr |  |  |  |  |  |  |  |
| Minimum number of individuals (MNI) | | | | |  | 1 | 16 | 3 | 12 | 20 | 11 | 1 | 4 | 7 | 1 | 2 | 1 | 33 | 8 | 1 | 1 | 1 | 1 | 1 | 6 | 2 |

**Table S2| biometric measurements of three *Vicia faba* from EWT**. Each measure was taken using Xradia Micro-CT-400 scan. *Distance calculated from the top of the seed to the end of the radicle.

|  | **Seed** | | | | | **Hilum** | |
| --- | --- | --- | --- | --- | --- | --- | --- |
| **Specimen** | **Volume (mm3)** | **Circumference (mm)** | **Position of the radicle* (mm)** | **Width** | **Length** | **Width** | **Length** |
|  |  |  |  |  |  |  |  |
| O9a_Basket 47 | 34.2 | 12.0 | 3,1 | 3,3 | 5,2 |  |  |
| P7d-Basket 123a | 33,8 | 12,1 | unknown | 3,3 | 4,9 | 0,7 | 2,6 |
| P7d-Basket 123b | 47,8 | 14,1 | 3,1 | 4,2 | 5,2 |  |  |

**Table S3| Variables selected for Principal Component Analysis (PCA).** All the values, except those of *V. faba*, refer to modern uncharred specimens. §archaeological specimens: °measured value of the charred *V. faba*, ¥ value of the circumference of the uncharred legume estimated according to **Caracuta *et. al*** (9) ^maximum circumference; *length as % of the circumference; seed outline: 1=heart-shaped; 2=sub-triangular; 3=ovate; 4=sub-lenticular; 5= sub-spherical; 6: sub-rectangular; hilum outline: 1=circumlinear, 2=linear; 3=oblong; 4=wedge; 5=oval.

|  | **Seed** | | **Hilum** | | |
| --- | --- | --- | --- | --- | --- |
|  |  | |  | | |
| ***Vicia*** | **Circumference^ (mm) (±SD)** | **outline** | **Absolute length (mm)** | **relative length* (%)** | **Outline** |
| **Modern varieties** |  |  |  |  |  |
| *articulata* | 16,0 (1,5) | 4 | 0,5 | 3 | 2 |
| *dasycarpa* | 14,2 (1,3) | 2 | 1,5 | 11 | 3 |
| *disperma* | 14,2(1,0) | 5 | 1,2 | 9 | 3 |
| *disperma*_a | 14,2 (1,0) | 6 | 1,0 | 9 | 3 |
| *eriocarpa* | 15,0 (1,0) | 5 | 1,5 | 10 | 2 |
| *eriocarpa*_a | 15,0 (1,0) | 5 | 1,5 | 10 | 3 |
| *ervilia* | 16,6 (0,5) | 2 | 1,2 | 7 | 5 |
| *ervilia*_a | 16,6 (0,5) | 6 | 1,2 | 7 | 5 |
| *galeata* | 16,5 (1,2) | 3 | 4,5 | 27 | 4 |
| *galeata*_a | 16,5(1,2) | 6 | 4,5 | 27 | 4 |
| *galilea* | 17,0 (1,0) | 5 | 2,5 | 15 | 5 |
| *hybrida* | 15,5(0,7) | 3 | 2,0 | 13 | 5 |
| *hybrida*_a | 15,5(0,7) | 5 | 2,0 | 13 | 5 |
| *johannis* | 16,7(1,2) | 5 | 2,0 | 12 | 5 |
| *lutea* | 17,0 (1,0) | 3 | 2,0 | 12 | 5 |
| *lutea*_a | 17,0 (1,0) | 5 | 2,0 | 12 | 5 |
| *macrocarpa* | 17,0 (0,5) | 3 | 2,0 | 12 | 3 |
| *macrocarpa*_a | 17,0 (0,5) | 5 | 2,0 | 12 | 3 |
| *narbonensis* | 15,0 (1,0) | 5 | 2,5 | 17 | 3 |
| *narbonensis*_a | 15,0 (1,0) | 6 | 2,5 | 17 | 4 |
| *peregrina* | 15,0 (0,5) | 3 | 1,0 | 7 | 5 |
| *peregrina*_a | 15,0 (0,5) | 5 | 1,0 | 7 | 5 |
| *palaestina* | 14,4 (1,0) | 2 | 2,3 | 16 | 5 |
| *palaestina*_a | 14,4 (1,0) | 3 | 2,3 | 16 | 5 |
| *palaestina*_b | 14,4 (1,0) | 5 | 2,3 | 16 | 5 |
| *pannonica* | 14,0 (1,2) | 2 | 2,0 | 14 | 5 |
|  |  |  |  |  |  |
|  |  |  |  |  |  |
|  |  |  |  |  |  |

**Continues from previous page. Table S3**

|  | **Seed** | | **Hilum** | | |
| --- | --- | --- | --- | --- | --- |
|  |  | |  | | |
| ***Vicia*** | **Circumference^ (mm) (±SD)** | **outline** | **Absolute length (mm)** | **relative length* (%)** | **Outline** |
| ***pannonica_a*** | **14,0 (1,2)** | **5** | **2,0** | **14** | **5** |
| ***sativa*** | **16,0 (1,0)** | **5** | **2,5** | **15** | **3** |
| ***sativa_a*** | **16,0 (1,0)** | **5** | **2,5** | **15** | **4** |
| ***serratifolia*** | **15,5 (1,3)** | **5** | **2,0** | **13** | **5** |
|  |  |  |  |  |  |
| ***Archaeological specimens*** |  |  |  |  |  |
| ***§faba el-Wad*** | **12,8º; 15,2 ¥(1,2)** | **1** | **2,8** | **18** | **3** |
| ***§fabaAhihud*** | **15,0 º;17,6¥ (0,5)** | **1** | **3,0** | **17** | **3** |
| ***§faba Yiftahel*** | **12,0 º;14,6¥ (0,5)** | **1** | **2,4** | **16** | **3** |
| ***§faba N. Zippori*** | **12,6 º;14,7 ¥ (0,4)** | **1** | **2,5** | **17** | **3** |

**Movie S1:** 3D model of *Vicia faba* coming from Square O9a_Basket 47**.** The video starts showing the frontal view of the legume, with the radicle partially missing. Then, the legume is rotated 90° counterclockwise showing the right cotyledon. The rotation continues showing the back, the top and the bottom of the legume.

**Movie S2**: 3D model of *Vicia faba* coming from Square P7d_Basket 123a. The video starts showing the back of the legume. Then, the legume is rotated 90° counterclockwise, showing the right cotyledon. The rotation of the legume continues showing in order, the top and the left cotyledon and the bottom with the fingerprint of the hilum (the grey elongated spot in the center).

**Movie S3**: 3D model of *Vicia faba* coming from Square P7d_Basket 123b. The video starts showing the back of the legume. Then, the legume is rotated 90° clockwise, showing in order the left cotyledon, the top and the bottom of the seed.
